# The effectiveness of training physical therapists in pain neuroscience education on patient reported outcomes for patients with chronic spinal pain: a study protocol for a cluster randomized controlled trial

**DOI:** 10.1186/s12891-018-2269-2

**Published:** 2018-10-25

**Authors:** Elizabeth Lane, Julie M. Fritz, Tom Greene, Daniel Maddox

**Affiliations:** 1 Department of Physical Therapy & Athletic Training, University of Utah, 520 Wakara Way, Salt Lake City, UT 84108 USA; 2 College of Health, University of Utah, 520 Wakara Way, Salt Lake City, UT 84108 USA; 3 Department of Internal Medicine and Director, Population Health Research Study Design and Biostatistics Center, School of Medicine, University of Utah, 295 Chipeta Way, Salt Lake City, UT 84132 USA; 4 Department of Physical Therapy, Ivester College of Health Sciences, Brenau University, 500 Washington St. SE, Gainesville, GA 30501 USA

**Keywords:** Chronic pain, Pain neuroscience education, Cluster randomized trial, Low back pain

## Abstract

**Background:**

Chronic spinal pain affects many in the United States and is associated with rising healthcare costs - but not improved outcomes. Education and self-care promotion are hallmarks of the recommended approach for this condition. Pain Neuroscience Education (PNE) is a method of educating patients about the neurophysiology of pain that aims to reconceptualize pain from an indicator of damage to an interpretation of input signals by the brain and nervous system. PNE has shown efficacy in controlled situations when delivered by experts, but its effectiveness has not been investigated among trained clinicians in a pragmatic setting.

**Methods:**

A cluster randomized trial will randomly assign 16 clinic regions to either receive PNE training or continue with usual care. Patients with chronic neck or back pain will be enrolled to provide outcome data. Measures will be collected at baseline, 2 weeks, and 12 weeks. The primary outcome will be the Patient-Reported Outcomes Measurement Information System (PROMIS) Physical Function computer-adapted test (PF-CAT). Pre-specified statistical analyses will compare outcomes between clinic regions assigned to PNE treatment or usual care while using random effects to account for region-level clustering.

**Discussion:**

Pain Neuroscience Education has been shown efficacious for a variety of patient-centered outcomes for those with chronic pain, but it has not yet been investigated outside of controlled settings. This trial has the potential to promote PNE as a low-cost intervention for chronic spinal pain and affect physical therapy education.

**Trial registration:**

ClinicalTrials.gov identifier NCT03168165, registered May 30, 2017.

## Background

Chronic spinal pain is a very common and costly condition. It affects an estimated 46% of the total population, with low back pain (LBP) being the most prevalent condition [1]. Lifetime prevalence of spinal pain ranges from 54 to 80%, and the estimated healthcare costs for those with spinal pain are 57% higher than those without [2]. While many with acute spinal pain have a favorable prognosis, those who develop chronic pain often experience persistent poor health and place a large burden on the healthcare system [2, 3]. Despite increasing expenditures for spinal pain, there is evidence that the prevalence of chronic spine pain and associated disability are actually increasing [4].

With growing healthcare costs and mounting disability, there is increased demand for physical therapists to promote more effective self-management strategies for patients with chronic spinal pain. Education and motivation to engage in treatment are critical components of self-management. Effective self-management requires physical therapists to understand strategies to engage patients in their treatment and facilitate necessary behavior changes. Strategies to promote patient engagement and self-management behaviors and their theoretical underpinnings are not familiar to many physical therapists and may require additional training.

Several theories have been proposed to explain human motivation for behavior change [5, 6]. Self-determination theory (SDT) is a well-supported theoretical framework for understanding motivation and its role in behavior and behavior change [5–7]. According to SDT, autonomous motivation, or motivation that is internal to the individual, is most likely to foster the persistence and engagement necessary for changing behavior; this is in contrast to motivation arising from extrinsic sources (e.g, meeting expectations of a family member or receiving a tangible reward) [7].

In SDT, autonomous motivation is fostered by an individual’s sense of autonomy (feeling in control of one’s own behaviors), competence (gaining mastery of tasks), and relatedness (feeling a sense of belonging and attachment) [5, 8]. Health care practitioners who are able to support a patient’s sense of autonomy and promote competence and relatedness can enhance health outcomes [9]. The role of autonomous motivation and strategies to enhance relatedness and competence has been examined in other disciplines but has not been extensively evaluated in a physical therapy setting.

Competence, or self-efficacy, relates to the degree an individual feels they have control over their situation [7]. Pain self-efficacy specifically is defined as a patient’s confidence in his or her ability to tolerate pain and perform daily activities despite pain [10]. Higher self-efficacy has been related to improved health outcomes and motivation towards more active self-management strategies [11, 12]. Barriers to greater pain self-efficacy for many patients are misperceptions or misunderstandings about pain. In other words, if patients feel that pain is directly proportional to tissue injury or damage, they are likely to feel that they have less control over managing that pain. Education focused on helping patients re-conceptualize pain from an indicator of damage to an interpretation of input signals by the brain and nervous system can enhance pain self-efficacy [13].

Relatedness refers to the desire to feel connected to others [8]. In healthcare, relatedness is often described as therapeutic alliance, defined as a collaborative relationship between patient and healthcare provider [14, 15]. Therapeutic alliance has been shown to be an important moderator between exercise treatment and global perceived effect in patients with chronic LBP [16].

The strategy being tested in this study to improve patients’ autonomous motivation through enhanced pain self-efficacy and therapeutic alliance is pain neuroscience education (PNE). PNE is an education method used by physical therapists to help patients understand the biology, physiology, and psychosocial factors influencing their pain experience [17, 18], and to reconcile faulty cognitions and beliefs associated with persistent pain and disability [19]. PNE has been shown to have positive effects on pain [18, 20, 21]. This is consistent with the biopsychosocial model of pain [22]. Previous work has demonstrated the efficacy of PNE on disability [17, 18, 20, 23], psychological factors [17, 18, 24, 25], physical movement [17, 18, 21, 24, 25], beliefs [25, 26], and healthcare costs [27, 28] for a variety of conditions including chronic pain. The proposed mechanism of PNE is related to changes in patients’ conceptualization of the pain experience [29] - specifically concepts associated with fear [30, 31], knowledge, and beliefs of pain [18, 20, 30]. According to SDT, competence is a basic psychological need [8]. We believe that educating the patient to re-conceptualize pain is a strategy through which physical therapists can foster self-efficacy, build relationships with patients, and enhance autonomous motivation, thus promoting behavior change [32].

Our overall goal is to determine the effectiveness of providing physical therapists with PNE training on patient-centered outcomes (Physical Function and Pain Interference) for patients with chronic neck or back pain undergoing care by a physical therapist.

## Methods

### Design and setting

This is a two-arm, single-blind, cluster randomized control trial to take place in 16 clinic regions within a large private rehabilitation practice in Atlanta, Georgia and Birmingham, Alabama. Clinic regions are the unit of randomization. We will randomly assign clinic regions to either receive PNE training for physical therapists employed in clinics within the region or usual care (UC) - with no additional training for physical therapists working in the region clinics. The clinic region was chosen over a single clinic for the randomization unit to reduce the potential contamination that may occur when physical therapists work in more than one clinic within a region. Randomization of clinic regions will be stratified by clinic region size and a measure of the region’s clinical outcome for LBP. Clinic region size was based on the average number of new evaluations per week during the period February 2016 – February 2017 over the participating clinics within the designated clinic region. The region’s clinical outcome for LBP is based on the average rank of the region’s clinics among other clinics in the company based on the Oswestry Disability Index [33], a common outcome tool used for LBP. The participating clinics from which the patients will be enrolled have provided permission to carry out this study.

Physical therapists working in clinics in participating regions will receive either PNE training or no additional training based on the random assignment of their clinic region. Outcomes will be assessed on consenting patients with chronic neck or LBP treated within these clinics. We will recruit patients meeting our eligibility criteria who are scheduled for physical therapy evaluation at a clinic within a participating region. Because the intervention is given to physical therapists, potentially eligible patients are asked to consent to provide outcome measures for the study, consistent with a cluster-randomized design. Physical therapists will provide informed consent to participate in the project and participate in training procedures. Other than the training to provide PNE (or not), there will be no attempts to direct physical therapist treatment or duration of care in any way as a result of participation in this study.

### Participant inclusion criteria

Patient participants in this project must be age 18–75 at time of their first session with a physical therapist, their primary reason for seeking physical therapist care must be low back and/or neck pain, and they must meet the NIH definition of chronic spinal pain [34] (i.e., neck or back pain on at least half the days in the past 6 months). Patient subjects must not have received physical therapist care in the previous 6 months or spinal surgery in the previous 12 months, must not demonstrate evidence of “red flag” conditions (e.g., cauda equine syndrome, cancer, fracture, infection or systemic disease) that would require immediate referral to a medical provider, and must not be knowingly pregnant.

These criteria are designed to be pragmatic and recruit a representative group of patients with chronic spinal pain beginning physical therapist care, excluding only those individuals whose episode of care may be at least partially dictated based on age (very young or very old), recent surgery, pregnancy, or concerns about a red flag condition. We will track recruitment of patients into the project and reasons for ineligibility. Patients who do not meet the eligibility criteria or choose not to enroll will not have their physical therapist care impacted in any way. These patients are compensated $15 for each of the assessment time points completed (up to $45). This is dispensed in the form of an electronic gift card. Due to the low risk nature of this trial, a cover letter consent will be utilized instead of requiring a signed consent document.

Physical therapist participants will be recruited from clinics in the 16 participating regions. Participating physical therapists must not have previously received formal training in pain neuroscience education totaling more than 8 h of instruction. They must not plan to leave the company during the recruitment period or hold a position that requires that they see patients in both control and intervention clinic regions.

These criteria are designed to recruit a group of physical therapists without prior exposure to PNE who treat patients within a single recruitment region of the study. Physical therapists who do not meet these criteria or who choose not to participate will not have their employment impacted, and they may choose to participate in training along with their primary clinic affiliation. We anticipate that new physical therapists will join participating clinics during the course of this project. We will provide an interim training session during the recruitment period as an opportunity to train newly-hired physical therapists working in clinics randomized to receive PNE education and provide therapists and additional opportunity to join the study and receive training. We will not recruit patients from any physical therapist until he or she has consented to participate and has received the appropriate training. If a physical therapist moves within the company between clinic regions, we will discontinue recruiting patients seen by the therapist unless he or she moves to a region randomly assigned to the same group as his or her previous region. The physical therapists receive 16 continuing education units for completing the education course as an incentive for participating. The physical therapists in the control clinic will also be trained at the end of the project. Physical therapists will also be consented via a cover letter consent form due to the low risk nature of their participation.

### Participant recruitment and allocation

Patient participants for this study will be identified by front office staff when requesting to be scheduled for an initial physical therapist evaluation. We will supply the front office staff with a scripted message notifying the patient that they may be eligible to participate in a research project and ask permission for a researcher to contact them. A secondary method to offer participation in the study will use the clinics’ electronic medical scheduling system (Raintree Systems, Temecula, CA), which allows custom reports to identify those scheduled for initial examinations with a diagnosis of neck or back pain. Lists of patients who meet this initial eligibility will be emailed by the research team to inform them of their potential eligibility and provide an opportunity to opt-out of being contacted. The research team will call the patient after 1 day to explain the study if they do not opt-out. Informed consent will be obtained by email with a consent cover letter. A patient is considered enrolled in the study when he or she begins completing the online baseline measures.

All project data will be collected using REDCap, an electronic data management system. This allows all data to be directly entered electronically by all subjects, patient and physical therapists, at all time points. REDCap automated process sends an email with a patient-specific link to all questionnaires. This minimizes the risk of bias based on group allocation from the investigators and reduces burden on any clinic or study personnel.

### Study measures

At baseline, the patient will provide demographic information including age, sex, race/ethnicity, employment status, and general medical and current history of symptoms.

The primary outcome will be physical function (PF), assessed using computer-adaptive test methods (CAT) developed by the Patient-Reported Outcomes Measurement Information System (PROMIS). PROMIS is an NIH-sponsored project using advanced psychometrics to develop patient-reported outcome measures with improved reliability, validity, and responsiveness [35, 36]. Physical function assesses an individual’s capability to perform physical tasks. CAT methods maximize validity while minimizing patient burden [37]. The PF-CAT has excellent reliability and concurrent validity [38] and is highly responsive to change [39].

Secondary outcome measures to be collected will include the PROMIS Pain Interference CAT, which assesses the consequences of pain on daily life. The Pain Interference CAT is highly reliable and validity based on correlation with other validated tools [38]. The Pain Self-Efficacy Questionnaire (PSEQ) quantifies an individual’s confidence in performing activities despite pain [10]. The Treatment Self-Regulation Questionnaire (TSRQ) is a self-report questionnaire developed to assess the degree of extrinsic or intrinsic (autonomous) motivation for engaging in healthy behavior - including beginning a treatment program [40]. The Working Alliance Theory of Change Inventory (WATOCI) is a 9-item tool that measures the strength of the alliance between a patient and therapist [41].

Other measures to be collected in the study will include potential covariates. The Numeric Pain Rating Scale (NPRS) ranges from 0 (no pain) to 10 (worst imaginable pain) as a measure of pain intensity. The Pain Catastrophizing Scale (PCS) is used to quantify catastrophic thoughts regarding pain. Scores range from 0 to 52, with higher scores indicating higher levels of pain catastrophizing [42]. The Global Rating of Change (GROC) is used to gain the patient’s perceived progress of their condition since the beginning of physical therapist care. The GROC ranges from + 7 (a very great deal better to 0 (about the same) to) -7(a great deal worse) [43]. The Neurophysiology of Pain Questionnaire (NPQ) is used to gain information regarding the how the patient conceptualizes the origins of his/her pain [30]. The schedule of collecting these measures is shown in Table 1.

**Table 1:** Schedule of patient self-reported measures

Measure	Baseline	2 Weeks	12 Weeks
PROMIS Physical Function	X	X	X
PROMIS Pain Interference	X	X	X
NPRS	X		X
PSEQ	X	X	X
TSRQ	X	X	X
PCS	X		X
WATOCI		X	X
GROC			X
NPQ	X	X	

*PROMIS* Patient-Reported Outcomes Measurement Information System*, NPRS* Numeric Pain Rating Scal*, PSEQ* Pain Self-Efficacy Questionnaire, TSRQ Treatment Self- Regulation Questionnaire*, PCS* Pain Catastrophizing Scale*, WATOCI* Working Alliance Theory of Change Inventory*, GROC* Global Rating of Change*, NPQ* Neurophysiology of Pain Questionnaire

### Pain neuroscience education training intervention

Physical therapists working in clinic regions randomized to receive PNE education will be trained using both in-person and online content. Latimer and colleagues [44] used a 16-h pain science education module training program with physical therapy students and were successful in changing the students’ beliefs and attitudes. A shorter (3-h) training strategy failed to show an effect on beliefs and attitudes [45]. Our study will provide a training dosage designed to replicate the 16-h program with a portion of the education taking place online, but supplemented with in-person practice designed to improve carryover into clinical practice. The online content (Table 2) will include the majority of the didactic portion of the training. The 6-h in-person session is designed to accomplish the following objectives: 1) *review the most important concepts from online training; 2) demonstrate incorrect* vs *effective PNE approaches using actual case examples, 3) practice proper assessment and PNE implementation using role-playing, and 4)* Discuss how to implement a PNE approach in conjunction with the therapists’ current therapeutic approaches of manual therapy and exercise.

**Table 2:** Online content for PNE training

Module 1	○ Epidemiology and Economic Impact of Chronic Pain ○ Historical Review and Update of the Understanding of Pain
Module 2	○ Assessing for Neurophysiologic and Neuromusculoskeletal Sources of Pain ○ Assessing for Faulty Beliefs and Behaviors: Use of questionnaires, questions to ask, etc.
Module 3	○ Review of Evidence for Conservative Strategies to Address Pain ○ Review of Evidence for PNE
Module 4	○ Practical Implementation of PNE - Part 1: PNE Concepts and Strategy for Assessment
Module 5	○ Practical Implementation of PNE - Part 2: How to differentiate different types of pain (Central sensitization, peripheral neuropathic, peripheral nocioceptive)
Module 6	○ Practical Implementation of PNE - Part 3: Application of PNE approach in conjunction with current approaches of exercise and manual therapy. ○ PNE Review and Practical Integration with Other Conservative Strategies

The total education content will be approximately 16 h. Physical therapists will be required to complete the online modules, attend the 6-h in-person practice session, score > 90% on the Neurophysiology of Pain Questionnaire, and score > 90% on a written course examination. A portion of the in-person session will be used to provide instructions in administrative aspects of the study.

### Fidelity monitoring

In order to monitor fidelity of the PNE provided by physical therapists in the training group, we will ask patients consenting to participate if their physical therapist provided education that equated pain with a threat and not an indicator of injury or damage at the 2-week assessment. This question will help to assess if PNE was provided in a manner that the patient recalled. We recognize the potential bias of patients not recalling that PNE was actually provided; however, we chose this strategy because if a patient is unable to remember the primary message of PNE after only 2 weeks, it is unlikely that it was provided in a manner likely to impact outcomes. In addition, the Neurophysiology of Pain Questionnaire will also be given to patients at baseline and at the 2-week time point to test patients’ knowledge of pain mechanisms. This will allow further exploration of our PNE training strategy’s ability to improve patient understanding of pain. Bi-weekly emails will be sent to the PNE-trained physical therapists highlighting an aspect from their training to encourage fidelity and maintenance of implementation of the PNE approach.

### Statistical methods

Pre-treatment baseline patient and clinic characteristics will be compared between randomized groups to assess chance imbalances. If differences are found, these variables may be included as covariates in sensitivity analyses. In accordance with the intentionto-treat principle, all patients will be analyzed in the group to which their physical therapist was randomized regardless of compliance or fidelity. A “per-protocol” secondary analysis will be considered if fidelity to providing PNE is a concern.

For the primary outcome (PF-CAT), we will apply a linear mixed model to compare the primary PROMIS PF-CAT score at 12 months between the randomized groups while including the baseline PROMIS PF-CAT as a fixed effect covariate, with nested random effects for provider and clinic region to account for clustering by provider and the clinic regions. If substantial imbalances are detected in patient factors at baseline, these factors will be added as additional covariates in sensitivity analyses.

Statistical significance will be evaluating using a 2-sided α = 0.05 for both primary and secondary outcomes without adjustment for multiple comparisons. Intent-to-treat principles will be used for the analysis with all participants analyzed based on the treatment group of the clinic in which they received care - regardless of the therapist’s compliance with providing the PNE intervention. Sensitivity analyses based on fidelity in providing PNE or based on spinal region (neck or low back) may be considered. In addition, we will explore between-group differences from baseline to 2 weeks to examine immediate effects of treatment by a physical therapist trained to provide PNE.

### Sample size and power calculations

We determined our sample size in order to detect a clinically relevant important effect on the PROMIS PF-CAT after 12 weeks with 80% power while accounting for clustering due to clinic regions. The literature on minimum clinically important differences for PROMIS scores is limited for spinal pain patient populations. In one study of 170 patients with LBP receiving spinal injections, Schalet [46] reported a difference in the mean T-score change of 4.5 vs. 1.7 on the PROMIS PF-CAT for patients reporting they were “better” vs. “worse” with a pooled change score SD of 6.4 (standardized effect size d = 0.43) [46]. Due to the lack of published data on meaningful effect sizes. we examined data from our own healthcare system and found a difference in T-scores for PROMIS PF-CAT of 4.2 vs. 0.72 for patients who reported improved vs. stable back pain based on concurrent Oswestry scores with a pooled SD = 6.9 (standardized effect size d = 0.50). Based on these studies, we will power this study to detect an effect size of d = 0.40. Based on reviews of past cluster randomized trials conducted in primary care settings, we assume an intra-cluster correlation of 0.025 [47, 48] to account for clustering of our primary outcome between the 16 clinic regions. We also assume that the primary outcome will be ascertained in 90% of enrolled patients. Under these assumptions, our cluster randomized design with 316 enrolled patients will provide approximately 80% power with 2-sided α = 0.05 to detect a mean difference in the PROMIS PF-CAT between clinic regions assigned to the PNE and usual care interventions. This calculation does not account for the savings in power that will result from adjusting for the baseline level of the outcome, and is therefore likely to be slightly conservative.

## Discussion

Chronic spinal pain is a challenging condition to treat for physical therapists. Pain Neuroscience has shown efficacy in trials with high internal validity [17, 18, 20, 23–27]. This study will use a more pragmatic approach to evaluate the effectiveness of providing PNE to physical therapists based on the impact on patient-centered outcomes. We have conceptualized the potential benefits of PNE from an SDT model, hypothesizing that PNE may benefit patients by enhancing autonomous motivation through a greater sense of relatedness to their physical therapist and their degree of pain self-efficacy.

While PNE has been shown efficacy in more controlled studies with greater internal validity [17, 18, 20, 23–28], effectiveness in a large setting with a more representative patient and provider population has not been explored. We have grounded our work in the Self-Determination Theory and collected many outcomes relevant to that theory. This would allow for further investigation into mechanisms of PNE in relation to SDT concepts in secondary analyses, such as autonomous motivation and self-efficacy.

One limitation of this study, which is common to pragmatic studies, is balancing internal validity with the need for greater generalizability. We will monitor fidelity to the intervention by querying patients about the key message of PNE, but a more robust fidelity assessment (e.g., videotaping of encounters, direct observation, etc.) would diminish generalizability of our results. Consistent with a pragmatic approach, we chose a cluster-randomized design to minimize the potential bias due to contamination from physical therapists with different study-related training working in the same clinic. We chose to randomize clinic regions instead of individual clinics because it is not uncommon for a physical therapist to work in more than one clinic within a region. While this design minimizes concerns for contamination, it reduces the number of units available for randomization and therefore required a greater number of patient participants in the study to obtain adequate statistical power. The researchers only interact with study participants in our study through email and phone correspondence. This could have an effect on study retention, but is consistent with a pragmatic approach.

## Abbreviations

GROC: Global Rating of Change
LBP: Low back pain
NPQ: Neurophysiology of Pain Questionnaire
NPRS: Numeric Pain Rating Scale
PCS: Pain Catastrophizing Scale
PF-CAT: Physical Function computer-adapted test
PNE: Pain Neuroscience Education
PROMIS: Patient-Reported Outcomes Measurement Information System
PSEQ: Pain Self-Efficacy Questionnaire
SDT: Self-determination theory
TSRQ: Treatment Self-Regulation Questionnaire
UC: Usual care
WATOCI: Working Alliance Theory of Change Inventory
